# Cross-sectional study comparing smart insoles and manual methods for short physical performance battery in hip fracture patients

**DOI:** 10.1007/s40520-025-02960-6

**Published:** 2025-03-01

**Authors:** Shinjune Kim, Soojin Kim, HyeonSu Kim, HyunBin Kim, Jun-Il Yoo

**Affiliations:** 1 Department of Orthopaedic Surgery, Bumin Medical Center, Seoul, Republic of Korea; 2 Department of Biomedical Research Institute, Inha University Hospital, Incheon, Republic of Korea; 3 Department of Kinesiology, Inha University, Incheon, Republic of Korea; 4 Department of Orthopedic Surgery, School of Medicine, Inha University Hospital, Inha University, 27, Inhang-ro, Jung-gu, Incheon, Republic of Korea

**Keywords:** Short physical performance battery (SPPB), Physical function, Hip fracture, Smart insoles

## Abstract

**Introduction:**

The Short Physical Performance Battery (SPPB) is a widely used tool for assessing physical function in older adults, including those who experienced a hip fracture. Traditionally, medical professionals perform the SPPB manually, which is time-consuming and prone to subjective interpretation. However, recent technological advancements have introduced smart insoles that can automatically capture and analyze data related to gait and balance, potentially offering a more objective and efficient method for performing the SPPB.

**Methods:**

In this study, we aimed to compare the smart insole method versus the manual method for SPPB assessment in hip fracture patients. We recruited a sample of 57 patients with hip fracture aged 50 years or older. The participants underwent both the smart insole assessment and the manual assessment simultaneously. The SPPB consists of three subtests: balance, gait speed, and chair stands.

**Results:**

The balance test scores slightly increased with smart insoles, showing a mean difference of 0.086 and a p-value of 0.037. In contrast, chair stand and gait speed tests showed negligible differences, with p-values of 0.777 and 1.000, respectively. The overall SPPB scores were closely matched between the methods, with a minimal mean difference of 0.103 and a p-value of 0.282. High correlations were observed across the assessments, with *r* of 0.95 for individual tests and 0.98 for total SPPB scores.

**Conclusion:**

The smart insole method provides a reliable and efficient alternative to the manual method for assessing SPPB in hip fracture patients. Smart insoles in assessments can save time and resources while improving accuracy and standardization of SPPB measurements.

## Introduction

Hip fractures are a major health concern among older adults, with an estimated annual incidence exceeding 1.6 million worldwide. These fractures can lead to mortality, and hip fracture patients often experience significant functional decline. Recovery and rehabilitation are further slowed by sarcopenia. Therefore, accurate and efficient assessment of physical function in hip fracture patients is crucial [1]. 

The Short Physical Performance Battery (SPPB) is a comprehensive and well-established tool used to assess physical function in older adults, particularly in those recovering from a hip fracture [2]. It comprises three distinct subtests: balance, gait speed, and chair stands, each evaluating crucial aspects of physical mobility [3, 4]. The balance test, for example, is designed to assess an individual’s stability in various standing positions—side-by-side, semi-tandem, and tandem—providing a nuanced view of their balance abilities. The gait speed test, typically over a distance of 4 m, quantifies the time taken to walk this length, serving as a proxy for overall walking ability and endurance. The chair stands test, on the other hand, evaluates the strength and coordination necessary to rise from a seated position without the use of arms, reflecting lower body strength. Therefore, SPPB scores are widely used to assess sarcopenia and fracture risk in older adults [5]. In the SOMMA study, muscle composition analysis indicates the results of objective functional assessments such as SPPB as follows: A recent study from AMRA Medical utilizing MRI-based muscle composition markers demonstrated a strong correlation between muscle fat infiltration (MFI), muscle mass z-score (MVz) and functional capacity in older adults [6]. 

Traditionally, these tests are administered manually by trained healthcare professionals [7]. However, this approach can be time-consuming and is inherently subjective, relying heavily on visual observation and manual timing [8]. This subjectivity can lead to variability in results, influenced by the skill and judgment of the observer [8, 9]. With the aging population increasing and the corresponding demand for timely and accurate physical function assessment, the need for a more objective and efficient method is clear [10, 11]. 

Smart insoles are emerging as a potential technological solution to overcome the limitations of the manual SPPB method [12]. These insoles are embedded with advanced sensors capable of capturing detailed data on gait and balance parameters such as step length, cadence, and stride velocity [13]. This data, wirelessly transmitted to a computer or mobile device, can be analyzed by specially designed algorithms to objectively quantify an individual’s physical function in the context of the SPPB.

Adopting smart insoles for SPPB assessment offers several advantages. Firstly, it removes the subjective element of manual observation and timing, thereby reducing the risk of human error and enhancing the accuracy of the measurements [13]. This leads to a more standardized approach to SPPB assessments, ensuring consistency regardless of who conducts the test or where it is performed. Secondly, the efficiency of smart insoles potentially saves time and resources by providing quicker completion of the SPPB compared to the manual method [14]. Moreover, the automated analysis eliminates the need for manual data interpretation [13]. Finally, the integration of technology in the assessment process may increase patient engagement and satisfaction, offering a more interactive and contemporary approach to evaluating physical function.

In light of these potential benefits, the purpose of this study is to compare the efficacy and practicality of the smart insole method to the traditional manual method for performing the SPPB, specifically in patients undergoing hip fracture. The goal is to establish the generalizability and effectiveness of smart insoles in a broad context before applying and evaluating them in the more specialized setting of hip fracture recovery. This approach will enable us to assess the reliability and feasibility of incorporating smart insoles into routine clinical practice for diverse patient groups. The findings are expected to shed light on both the benefits and challenges associated with the implementation of smart insoles in physical function assessment, guiding the development of targeted applications for various patient demographics.

## Methods and materials

### Study design

This study employs a cross-sectional design to compare the smart insole method with the manual method for SPPB measurement and assessment of physical function in patients who have undergone hip fracture. Also, the study aims to recruit a sample of 57 hip fracture patients aged 50 and older. Participants will be selected from orthopedic clinics.

#### Participants

Inclusion criteria include a history of hip fracture, the ability to walk with or without assistive devices, and the ability to understand and follow instructions for the assessments. Exclusion criteria include cognitive impairment severe enough to interfere with participation, and any musculoskeletal or neurological condition that significantly affects gait and balance.

#### Ethical considerations

The study adhered to the principles outlined in the Declaration of Helsinki and received approval from the Institutional Review Board at Inha University Hospital. All research procedures were conducted in strict compliance with ethical standards, ensuring the protection of participants’ privacy, confidentiality, and rights.

### Smart insole

The smart insoles used in this study are the SALTED Smart insole (SALTED, Seoul, Republic of Korea), commercially available devices equipped with sensors to measure gait parameters such as step length, cadence, and stride velocity. The insoles will be placed into standardized indoor shoes provided by the hospital. The sensor data will be wirelessly transmitted to a computer or mobile device for analysis. The insoles will be calibrated and synchronized with the analysis software prior to each assessment.

### SPPB assessment

Participants were instructed to undergo three tasks: standing balance, a 4-meter walk, and chair stand test [4]. For the standing balance test, participants were required to maintain three different positions - feet side by side, semi tandem, and tandem. A score of 1 was assigned if the side by side (feet parallel) was sustained for 10 s, while a score of 0 indicated inability to maintain it. Semi tandem (heel side of one foot touching the big toe of the other foot) was assigned 1 point if sustained for 10 s, while failure to maintain it resulted in 0 points. Tandem stance (placing the heel of one foot directly in front of the other to achieve full alignment) was scored as follows: 3 points for maintaining the stance for over 10 s, 1 point for sustaining it for more than 3 s but less than 10 s, and 0 points for an inability to maintain the stance for more than 3 s. Subjects chose the foot placed in front based on personal comfort. Measurements ceased if the subject lost balance or moved their feet.

The gait speed test assesses the time taken to traverse a 4-meter distance at one’s typical walking pace. Participants are instructed to walk as they normally would. They received a score of 4 points for completing the task within 4.82 s or less, 3 points for completing it between 4.82 and 6.20 s, 2 points for finishing between 6.21 and 8.7 s, 1 point for exceeding 8.7 s, and 0 points for being unable to walk. Two measurements were conducted, and the fastest time recorded was used for evaluation.

The chair stand test assesses the time required for participants to complete five repetitions of standing up from and sitting back down on a chair, with arms folded across the chest, as rapidly as possible. Researchers recorded the duration from the verbal cues “ready, start” until the participant finished the fifth stand. Scoring is as follows: 4 points for durations less than 11.2 s, 3 points for durations between 11.2 and less than 13.7 s, 2 points for durations between 13.7 and less than 16.7 s and 1 point for durations exceeding 16.7 s. If the test cannot be completed within 60 s or is discontinued due to safety concerns, it is halted, resulting in a score of 0 points.

### Statistical analysis

Statistical analysis was conducted to compare the results obtained from the smart insole method and the manual method. Descriptive statistics were used to characterize the demographic features of hip fracture patients. To compare the smart insole and manual measurements, difference testing and correlation analysis were employed. Specifically, for continuous variables such as the measurement time, the difference was tested using either a paired t-test or a Wilcoxon signed-rank test, depending on whether the data satisfied the criteria for normality. Additionally, Pearson’s correlation analysis was used to evaluate the linear relationship between the two measurement values. For the SPPB scores, categorized according to the SPPB criteria, the association was assessed using Wilcoxon signed rank test, as these scores represent categorical variables. Similarly, Kendall’s correlation analysis was used to evaluate the linear relationship. The analyses were conducted using R Studio, and the ggplot2 library was utilized for data visualization. To assess whether the type of surgical procedure (intramedullary nail vs. prosthesis) influenced the SPPB results, we conducted a subgroup analysis. Furthermore, to ensure a homogeneous study population in terms of functional mobility, we only included patients with a Koval score of 2 or higher at the time of assessment.

## Results

The demographic profile of the participants in this study is detailed in Table 1. A total of 57 subjects were included, comprising 18 males and 39 females. The average age of the participants was 70.53 years, with a variation of ± 11.47 years. Regarding physical characteristics, the average height was 158.96 cm (± 10.33 cm), and the average weight was 60.28 kg (± 14.63 kg). The body mass index of the subjects averaged 23.60 kg/m² (± 3.80 kg/m²). Additionally, the average grip strength was found to be 18.75 kg (± 10.78 kg).

**Table 1:** Demographic characteristics of subjects used in this study

Characteristics	Total (*n* = 57)
Gender, n	Male: 18, Female: 39
Age, years	70.53 $\:\pm\:$ 11.47
Height, cm	158.96 $\:\pm\:$ 10.33
Weight, kg	60.28 $\:\pm\:$ 14.63
BMI, kg/$\:{\text{m}}^{2}$	23.60 $\:\pm\:$ 3.80
Grip strength, kg	18.75 $\:\pm\:$ 10.78

**Fig. 1:**
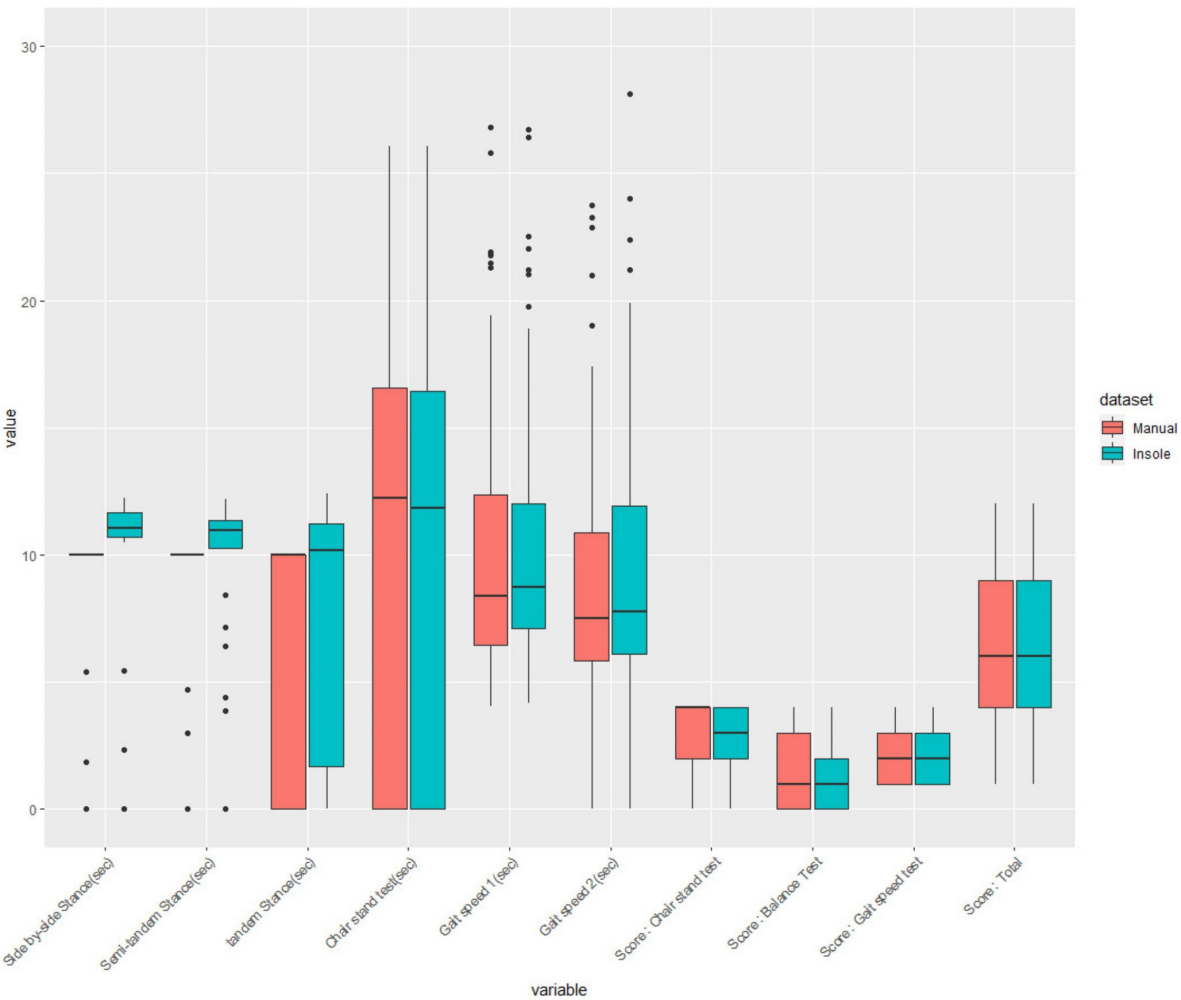
Distribution of SPPB assessment scores by manual and smart insole methods

Table 2; Fig. 1 provide a detailed comparison between the SPPB assessments performed using manual methods and those obtained via smart insole technology. The Side-by-side Stance assessment recorded an average score of 9.870s (± 3.690) for the manual method, contrasting with 8.746s (± 3.234) obtained via the smart insole method, resulting in a significant mean difference of -1.124s (± 0.658) and a p-value of < 0.001, indicating a considerable discrepancy between the two assessment techniques. Similarly, the Tandem Stance showed a mean score of 6.850s (± 5.047) for the manual assessments versus 6.136s (± 4.569) for the smart insole assessments, with a mean difference of -0.714s (± 1.063) and a p-value of < 0.001, reinforcing the trend observed in the Side-by-side Stance. The Semi-tandem Stance presented average scores of 9.050s (± 4.225) for the manual method and 8.408s (± 3.580) for the smart insole method, with a less pronounced but still significant mean difference of -0.642s (± 1.766) and a p-value of 0.008. This suggests a consistent pattern where manual methods tend to yield slightly higher performance scores compared to the smart insole in balance test scores.

**Table 2:** SPPB assessment using manual and smart insole methods

	Mean_M	SD_M	Mean_I	SD_I	Mean_diff	SD_diff	*p*-value
Side by-side Stance	9.870	3.690	8.746	3.234	-1.124	0.658	0.000***
Semi-tandem Stance	9.050	4.225	8.408	3.580	-0.642	1.766	0.008**
Tandem Stance	6.850	5.047	6.136	4.569	-0.714	1.063	0.000***
Chair stand test	11.510	9.547	11.586	9.783	0.076	1.369	0.674
Gait speed (trial1)	10.602	5.622	10.337	5.685	-0.265	1.766	0.257
Gait speed (trial 2)	9.635	5.587	9.318	5.393	-0.317	2.042	0.242
Score: Balance Test	2.810	1.444	2.897	1.459	0.086	0.283	0.037*
Score: Chair stand test	1.483	1.328	1.500	1.354	0.017	0.350	0.777
Score: Gait speed test	1.966	1.059	1.966	0.991	0.000	0.324	1.000
Score: Total	6.276	3.232	6.379	3.133	0.103	0.667	0.282

Mean_M and SD_M: Mean and standard deviation of values measured by the manual method.; Mean_I and SD_I: Mean and standard deviation of values measured by the smart insole method.; Mean_diff and SD_diff: Mean and standard deviation of the differences between the manual method and smart insole method measurements.; ***: p-value < 0.001, **: p-value < 0.01, *: p-value < 0.05

On the other hand, the Chair Stand Test demonstrated a negligible mean difference between the two methods, with scores of 11.510s (± 9.547) for manual and 11.586s (± 9.783) for smart insole, and a p-value of 0.674, indicating no significant difference. The two trials of Gait Speed assessments also showed minimal differences, with p-values of 0.257 and 0.242, respectively, suggesting comparable outcomes between the two methodologies for these tasks.

Further analysis of the individual scores for balance, chair stand, and gait speed tests revealed a statistically significant slight increase in balance test scores when assessed with smart insoles (mean difference 0.086, p-value = 0.037), whereas the differences in chair stand and gait speed test scores were negligible and not statistically significant, with p-values of 0.777 and 1.000, respectively.

The overall comparison of the total SPPB scores demonstrates a minimal mean difference of 0.103 (± 0.667) between the manual and smart insole methods, with a p-value of 0.282. This indicates no significant difference and highlights the similar effectiveness of both methods in assessing physical performance. Although certain tasks, especially those evaluating stance stability, show some differences, the results for overall physical performance are closely aligned between the manual and smart insole methods.

**Fig. 2:**
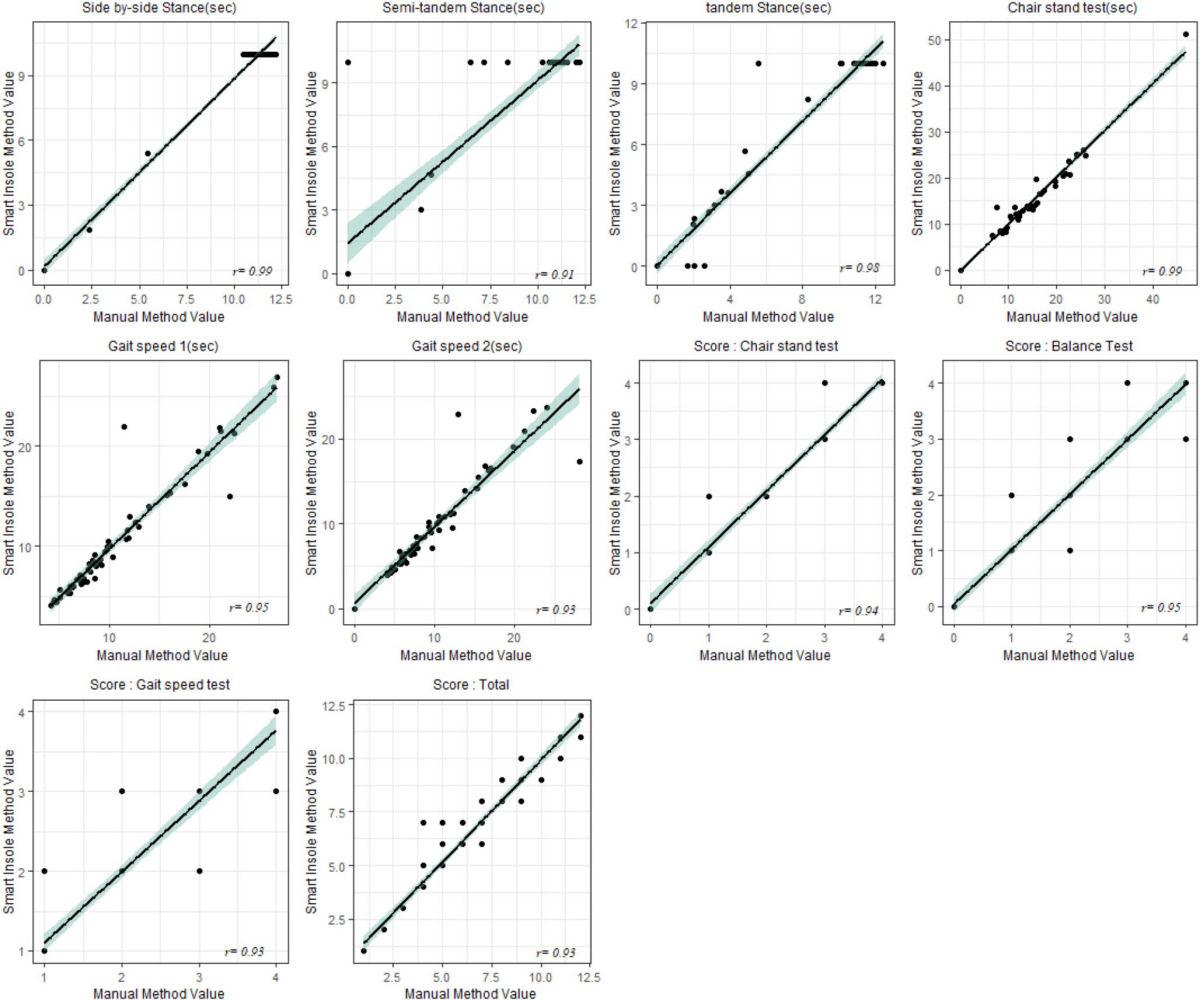
Correlation of SPPB assessment scores between manual and smart insole methods

Figure 2 illustrates this point through a series of scatter plots that compare scores from both methods across various SPPB assessments. The correlation coefficients are consistently high, indicating a strong linear relationship. Specifically, the Side-by-side Stance and Semi-tandem Stance are shown to have correlation coefficients of 0.99, indicating almost perfect positive linear correlations. The Tandem Stance, Chair Stand Test, and Balance Test are also highly correlated, each with a coefficient of 0.95. Similarly, the gait speed tests for both trials are correlated with a coefficient of 0.95. Lastly, the overall SPPB score exhibits a correlation of 0.98. These high correlation coefficients confirm the close agreement in scores from the manual and smart insole methods across various tasks measuring physical performance.

## Discussion

Hip fractures are a major health concern among older adults, with an estimated annual incidence exceeding 1.6 million worldwide. These fractures can lead to mortality, and hip fracture patients often experience significant functional decline. Recovery and rehabilitation are further slowed by sarcopenia. Therefore, accurate and efficient assessment of physical function in hip fracture patients is crucial.

Our study findings demonstrate that the smart insole method is highly correlated with the traditional manual method for SPPB measurement and assessment of physical function in hip fracture patients. This suggests that the smart insole method can be a reliable and valid alternative to the manual method for evaluating physical function in this population [13, 15–17]. 

The high correlation between the smart insole method and the manual method indicates that both methods are capturing similar aspects of gait and balance and are providing consistent measures of physical function. The agreement in determining the overall SPPB score as well as the individual subtest scores further supports the validity of the smart insole method. These results are promising and point towards the potential of implementing smart insoles in routine clinical practice for SPPB assessments in hip fracture patients [9, 18, 19]. 

The efficiency of the smart insole method is an additional advantage [20, 21]. The faster completion times compared to the manual method can save valuable time for healthcare professionals, allowing more efficient assessment of physical function in busy clinical settings. The shorter assessment time can also be beneficial for many patients, especially those who may have limited endurance or find the traditional assessment procedures tiring or burdensome [15, 16, 22]. Patient engagement and satisfaction are essential aspects of any assessment tool, and the positive feedback from participants suggests that the smart insole method can promote greater patient involvement in their own healthcare [12]. 

Overall, the smart insole method offers several advantages over the manual method, including enhanced objectivity, efficiency, standardization, and patient satisfaction. However, further research is needed to validate these findings in larger and more diverse populations. Longitudinal studies can investigate the reliability and stability of the smart insole measurements over time, as well as its sensitivity to changes in physical function. Additionally, cost-effectiveness studies can assess the economic feasibility of implementing this technology in routine clinical practice.

This study has several limitations. The small sample size and the exclusive focus on hip fracture patients may limit the generalizability of the findings. The cross-sectional design also prevents assessment of long-term outcomes. While the smart insole method showed high correlation with manual SPPB assessments, potential limitations exist in sensor accuracy and reliability. Factors such as variations in footwear and gait patterns may affect measurements. Additionally, the study does not evaluate the cost-effectiveness or feasibility of implementing smart insoles in clinical practice. Lastly, although SPPB is a simple and widely used test, the introduction of smart insoles may add complexity to its administration. Further research is needed to determine whether this technology enhances clinical utility or poses barriers to adoption.

## Conclusion

In conclusion, the smart insole method shows promise as a reliable and efficient alternative to the manual method for SPPB measurement and assessment of physical function in hip fracture patients. This technology has the potential to enhance clinical practice by providing objective and quantitative data on gait and balance parameters. Implementing smart insoles in routine assessments can save time and resources, while improving accuracy and standardization in SPPB measurements. Future studies should continue to explore the benefits and limitations of this technology in different populations and settings.

## Data Availability

No datasets were generated or analysed during the current study.
